# The outcomes of polyparasitism in stray cats from Brazilian Midwest assessed by epidemiological, hematological and pathological data

**DOI:** 10.1590/S1984-29612022033

**Published:** 2022-07-04

**Authors:** Alanderson Rodrigues da Silva, Gisele Braziliano Andrade, Joyce Katiuccia Medeiros Ramos Carvalho, Wanessa Teixeira Gomes Barreto, Filipe Martins Santos, Keyla Carstens Marques de Sousa, Marcos Rogério André, Luiz Claudio Ferreira, Rodrigo Caldas Menezes, Heitor Miraglia Herrera

**Affiliations:** 1 Programa de Pós-graduação em Biotecnologia, Universidade Católica Dom Bosco, Campo Grande, MS, Brasil; 2 Programa de Pós-graduação em Ciências Ambientais e Sustentabilidade Agropecuária, Universidade Católica Dom Bosco, Campo Grande, MS, Brasil; 3 Departamento de Medicina Veterinária, Universidade Católica Dom Bosco, Campo Grande, MS, Brasil; 4 Programa de Pós-graduação em Ecologia e Conservação, Universidade Federal de Mato Grosso do Sul – UFMS, Campo Grande, MS, Brasil; 5 Departamento de Patologia Veterinária, Universidade Estadual Paulista – UNESP, Jaboticabal, SP, Brasil; 6 Laboratório de Pesquisa Clínica em Dermatozoonoses em Animais Domésticos, Instituto Nacional de Infectologia Evandro Chagas – INI, Rio de Janeiro, RJ, Brasil

**Keywords:** Toxoplasma gondii, Platynosomum illiciens, Feline Immunodeficiency virus, Feline Leukemia virus, *in situ* hybridization, Toxoplasma gondii, Platynosomum illiciens, vírus da Imunodeficiência Felina, vírus da Leucemia felina, hibridização *in situ*

## Abstract

We evaluated the epidemiological, hematological, and pathological data of *Leishmania* spp., *Toxoplasma gondii*, *Platynosomum illiciens*, feline immunodeficiency virus (FIV), and feline leukemia virus (FeLV) infections and the coinfections in stray cats of an endemic area for leishmaniasis. The diagnosis was performed by serological tests and necropsy. We described gross lesions and histopathological findings. We used immunohistochemistry and chromogenic *in situ* hybridization for *L. infantum* detection. We found infection in 27 out of 50 sampled cats, among them, 14 presented coinfections. A strong correlation between splenomegaly and lymphadenomegaly with FeLV, and an association between hepatic lesions and cachexia with parasitism due to *P. illiciens* were observed. Moreover, we found a significant increase in the monocyte count in the FeLV-infected and a decrease in the red blood cell count in the FIV-infected animals. Amastigote forms of *Leishmania* spp. and tissue changes were detected in lymphoid organs of an animal coinfected with *P. illiciens*, *T. gondii*, and FIV. Polyparasitism recorded in stray cats of the Brazilian Midwest should be considered in effective control strategies for public health diseases. Moreover, stray cats of Campo Grande may be a source of infection of FIV, FeLV and *P. illiciens* for populations of domiciled cats.

## Resumo

Foi avaliada a epidemiologia, hematologia e patologia das infecções por *Leishmania* spp., *Toxoplasma gondii*, *Platynosomum illiciens,* vírus da imunodeficiência felina (FIV) e vírus da leucemia felina (FeLV) e das coinfecções em gatos não domiciliados em uma área endêmica para leishmaniose. O diagnóstico foi realizado por exames sorológicos e necropsia. Foram descritas lesões macroscópicas e achados histopatológicos. Usaram-se imuno-histoquímica e hibridização cromogênica *in situ* para detecção de *L. infantum*. Encontrou-se infecção em 27 dos 50 gatos amostrados, dentre eles, 14 apresentavam coinfecções. Foi observada forte correlação entre esplenomegalia e linfadenomegalia com FeLV, e associação entre lesões hepáticas e caquexia com parasitismo por *P. illiciens*. Além disso, foi encontrado um aumento significativo na contagem de monócitos nos animais infectados pelo FeLV e uma diminuição na contagem de hemácias nos animais infectados pelo FIV. Formas amastigotas de *Leishmania* spp. e alterações teciduais foram detectadas em órgãos linfoides de um animal coinfectado com *P. illiciens*, *T. gondii* e FIV. O poliparasitismo registrado em gatos errantes do Centro-Oeste brasileiro deve ser considerado nas estratégias de controle de zoonoses. Além disso, gatos errantes de Campo Grande podem ser fontes de infecção de FIV, FeLV e *P. illiciens* para populações de gatos domiciliados.

## Introduction

Parasitism is an interspecific relationship in which one species finds its ecological niche in another, including viruses and several eukaryotic organisms such as bacteria, protozoa, rickettsiae, helminths, and arthropods (Araújo et al., 2003). Different gradients of metabolic interdependence among parasites and their hosts may be observed over time (Lenzi & Vannier-Santos, 2005; Poulin, 2007). Moreover, the consequences of parasitism on the host’s health will vary according to parasitic load, concomitant infections, and some inherent characteristics of the hosts, such as age, gender, and nutritional and immunological status (Poulin & Combes, 1999).

Since parasites are globally spread in different ecological niches, representing approximately 40% of all biodiversity, it is expected that animals can be exposed to infections by several parasites simultaneously (coinfections) (Cox, 2001; Dobson et al., 2008; Viney & Graham, 2013). However, the results of coinfections in the health of their hosts are unpredictable due to the complexity of their interrelationships (Cox, 2001; Santos et al., 2018), mainly regarding parasites species that have a remarkable ability to suppress and/or modulate the immune response of their hosts, resulting in changes in pathogenicity, transmission, and virulence (Telfer et al., 2010; Alizon et al., 2013; Damania & Dittmer, 2014).

Although cats are not considered key hosts for *Leishmania infantum* infection, these domestic species have been reported to be naturally parasitized in endemic areas worldwide (Martín-Sánchez et al., 2007; Pimenta et al., 2015; Iatta et al., 2019; Asgari et al., 2020; Baneth et al., 2020), as well as in Brazil (Mello, 1940; Passos et al., 1996; da Silva et al., 2008; Coelho et al., 2010; Vides et al., 2011; Silva et al., 2014; Metzdorf et al., 2017; Rocha et al., 2019). Furthermore, records indicated that cats parasitized by *Leishmania* spp. become more likely to coinfections by feline immunodeficiency virus (FIV), feline leukemia virus (FeLV), and *Toxoplasma gondii* (Dorny et al., 2002; Sobrinho et al., 2012; Spada et al., 2012; Poffo et al., 2017). The association between cats seropositive for *Toxoplasma gondii* and retrovirus has also been reported in other countries (Dorny et al., 2002; Maruyama et al., 2003; Dubey et al., 2009; Cong et al., 2016). This study aimed to assess the epidemiological, hematological, and pathological data of *Leishmania* spp., *T. gondii*, *Platynosomum illiciens*, FIV, and FeLV infections, and coinfections, in stray cats from the city of Campo Grande in Midwestern Brazil, an endemic area for human and canine visceral leishmaniasis (VL).

## Materials and Methods

### Animals and sample collection

We sampled 50 adult stray cats (*Felis catus*) from the Zoonotic Disease Control Center (CCZ) in the municipality of Campo Grande, Mato Grosso do Sul state, an endemic area for visceral leishmaniasis (VL) in Brazil (20º29’19”S, 54º36’20”W). These cats were randomly selected with no limitation for age, sex, and clinical status during January–June 2019. The CCZ is responsible for the development of actions aimed at the control of zoonosis through the control of populations of stray dogs and cats including euthanasia according CCZ protocol, in accordance with the ethical standards established by the Federal Council of Veterinary Medicine published in the Brazilian guide to good practices for euthanasia in animals (CFMV, 2020). This study was approved by the Ethics Committee for the Use of Experimental Animals of the Universidade Católica Dom Bosco (UCDB) (protocol number 004/2014).

After the 50 cats had been sedated, whole blood was collected by punction of the jugular vein and placed in tubes containing ethylenediamine tetraacetic acid. Red blood cell (RBC), and white blood cell (WBC) counts were performed using an automated cell counter (Sysmex poch 100iV®). The hematocrit value was detected using the microhematocrit technique, and specific leucometry was performed by counting 100 leukocytes in blood smears stained with Giemsa. Necropsies were performed in the Department of Pathology of UCDB with the purpose of evaluating tissue alterations, including cachexia as a parameter for assessing body condition, as well as the search for *P. illiciens*, an endemic Trematoda in the study area (Personal communication from Andrade, GB) in the liver parenchyma. After gross evaluation, tissue fragments of the lymph node, spleen, and liver were collected and fixed in buffered formalin (pH 7,3) for 24 h.

### Histopathological evaluation

Fragments of liver, spleen, and lymph nodes were processed using the usual histology slide preparation techniques and stained with hematoxylin and eosin (HE) for light microscopy analysis (Luna, 1968). Histopathology evaluated tissue damage, pattern of inflammation, and the presence of amastigotes of *Leishmania* spp.

### Serological diagnosis

For the detection of IgG antibodies to FIV (96% sensitivity and of 98% specificity) and FeLV p27 antigens (90.70% sensitivity and 97.78% specificity) (Medeiros et al., 2019) in whole blood, we used Alere FIV Ac and FeLV Ag Test Kit^®^ chromatographic immunoassays according to Feitosa et al. (2021) and in accordance with the manufacturer’s recommendation. The presence of *T. gondii* and *L. infantum* antibodies in the serum of sampled animal was detected by the Indirect fluorescent Assay (IFAT), as described previously (André et al., 2010; Oliveira et al., 2008). *Toxoplasma gondii* RH strain tachyzoites and promastigotes of *L. infantum* were used as antigens, ten microliters of sera at dilution of 1:40 (cut-off for *T. gondii*) and 1:40 (cut-off for *L. infantum*) were placed in wells on antigen slides. Cat serum samples positive and negative for *T. gondii* and *L. infantum* (André et al., 2010; Braga et al., 2012), obtained from the serum bank of the Laboratory of Immunoparasitology, Department of Veterinary Pathology of Universidade Estadual Paulista, Jaboticabal, São Paulo, were also used in the serological reactions. Slides were incubated at 37°C in a moist chamber for 45 min, washed three times in phosphate-buffered saline (pH 7.2) for 5 min, and air-dried at room temperature. Immunoglobulin G (IgG) anti-cat conjugate (whole molecule with fluorescein isothiocyanate, dilution of 1:32; SigmaR, St. Louis, Missouri) for domestic feline samples were diluted according to the manufacturer’s instructions and added to each well. These slides were incubated again, washed, dried, and overlaid with buffered glycerin (pH 8.7), covered with glass coverslips, and examined using an epifluorescence microscope (Olympus, Japan).

### Immunohistochemical technique for Leishmania spp.

Immunohistochemistry (IHC) for *Leishmania* spp. was performed in the lymphoid tissues of the five *Leishmania* spp. seropositive cats. Antigen retrieval was performed with citrate buffer and then incubated in Protein Block® (Thermo Fisher Scientific, Cheshire, UK). Sections of the spleen and lymph nodes were treated with rabbit polyclonal anti-*Leishmania* antibody (*in-house*) (Boechat et al., 2016). The EasyLink One HiDef HRP® detection system was used. The slides were counterstained with hematoxylin and observed under a light microscope. Positive and negative controls were included.

### In situ hybridization for Leishmania infantum

Chromogenic *in situ* hybridization (CISH) was used to identify if the amastigote forms detected in the five positive cats for *Leishmania* spp. by IHC were those of *L. infantum*. We used CISH in order to associate tissue reaction with the presence of the parasite. For this purpose, a *L. infantum*-specific oligonucleotide probe labeled with digoxigenin that targets the minicircle kinetoplast DNA gene of the parasite was used (Menezes et al., 2013). Five micrometer thick sections were cut from the paraffin blocks and mounted on silanized slides. These sections were processed as previously described (Boechat et al., 2016), using the ZytoFastPlus chromogenic CISH Implementation Kit AP-NBT/BCIP1 (Zytovision GmbH, Bremerhaven, Germany). The probe was diluted at 1:500 in the hybridization solution H7782 (Sigma-Aldrich Co., St. Louis, MO, USA). Positive and negative controls were included.

### Statistical analyses

The Shapiro-Wilk test was performed to verify the normality of the hematological results. When appropriate, the paired t-test (normal distribution) or paired Wilcoxon test (non-normal distribution) was used to compare the means of hematological variables among infected and non-infected animals. The level of significance was set at p <0.05. Generalized linear models (GLM) were used to investigate associations among infections and necropsy findings. The candidate models were compared using the corrected Akaike information criterion (AICc). The model with a lower AICc value was considered the best-fit model (Akaike, 1974).

## Results

Overall, we found that 54% (27/50) of the sampled cats presented infections, among them 51.8% (14/27) displayed coinfections and 48.1% (13/27) showed infections by retrovirus (six single and seven coinfections) (Table 1). The seroprevalence of *T. gondii*, FeLV, FIV, and *Leishmania* spp. was 28% (n=14), 18% (n=9), 12% (n=6), and 10% (n=5), respectively. Furthermore, during the necropsy we found *P. illiciens* in the hepatic parenchyma of 22% (n=11) of the sampled cats. Single infections by *T. gondii* and FeLV were observed in six (12%) and five (10%) of the sampled animals, respectively (Table 1). Moreover, we found one cat infected only with *Leishmania* spp. and one infected only with FIV (Table 1). With respect to coinfection with retroviruses (n=5), we found two cats coinfected with *T. gondii* and FeLV, two with FIV and *P. illiciens*, and one with FIV and FeLV. Two of the sampled cats showed coinfection with four infectious agents (Table 1), and in one of these cats *L. infantum* was detected by CISH (Table 1). We highlight that all cats parasitized with *P. illiciens* showed concomitant infections.

**Table 1:** Single infections and coinfections in stray cats of Campo Grande, Midwestern Brazil. Data are expressed by the number of positive samples following by percentage of occurrence.

**Infections**	**number of positives (% of occurence)**
*Toxoplasma gondii*	6 (12)
Feline Leukemia Virus	5 (10)
Feline Immunodeficiency Virus	1 (2)
*Leishmania* spp.	1 (2)
Total single infection	13 (26)
*Platynosomum illiciens + T. gondii*	5 (10)
*P. illiciens + Leishmania* spp.	2 (4)
FeLV + *T. gondii*	2 (4)
FIV + *P. illiciens*	2 (4)
FIV + FeLV	1 (2)
*Leishmania* spp. + *P. illiciens* + *T. gondii* + FIV	1 (2)
*Leishmania* spp. + *P. illiciens* + FIV + FeLV	1 (2)
Total coinfection	14 (28)
Negative	23 (46)
Total Sampled	50 (100)

The Shapiro-Wilk test showed that RBC and hematocrit were normally distributed, while monocyte, lymphocyte, neutrophil, eosinophil, and basophil counts did not display normal distribution. Moreover, our statistical analyses demonstrated a strong correlation between splenomegaly and lymphadenomegaly in FeLV seropositive cats (Table 2). In fact, at necropsy, 88.9% (8/9) of the FeLV seropositive cats presented these findings. Moreover, significant higher monocyte counts (p = 0.01) were detected in FeLV seropositive (2,160/μL) than seronegative animals (513/ μL). Furthermore, a significant decrease in RBC was found in FIV seropositive (6,182,500/μL) compared to seronegative (8,414,500/μL) cats (p<0.05).

**Table 2:** Associations among necropsy findings and *Platynosomum illiciens* and feline leukemia virus performed using generalized linear models (GLM) on 50 stray cats from Campo Grande, Mato Grosso do Sul, Brazil.

**Necropsy findings**	***Platynosomum illiciens***	**Feline Leukemia Virus**
Lymphadenomegaly		0.26 (0.03; 0.49)*
Splenomegaly		0.64 (0.34; 0.95)**
Cachexy	-0.24 (-0.46; -0.02)*	
Hepatopathy	0.29 (0.03; 0.55)*	

* P= 0.05;

** P= 0.001

**Figure 1:**
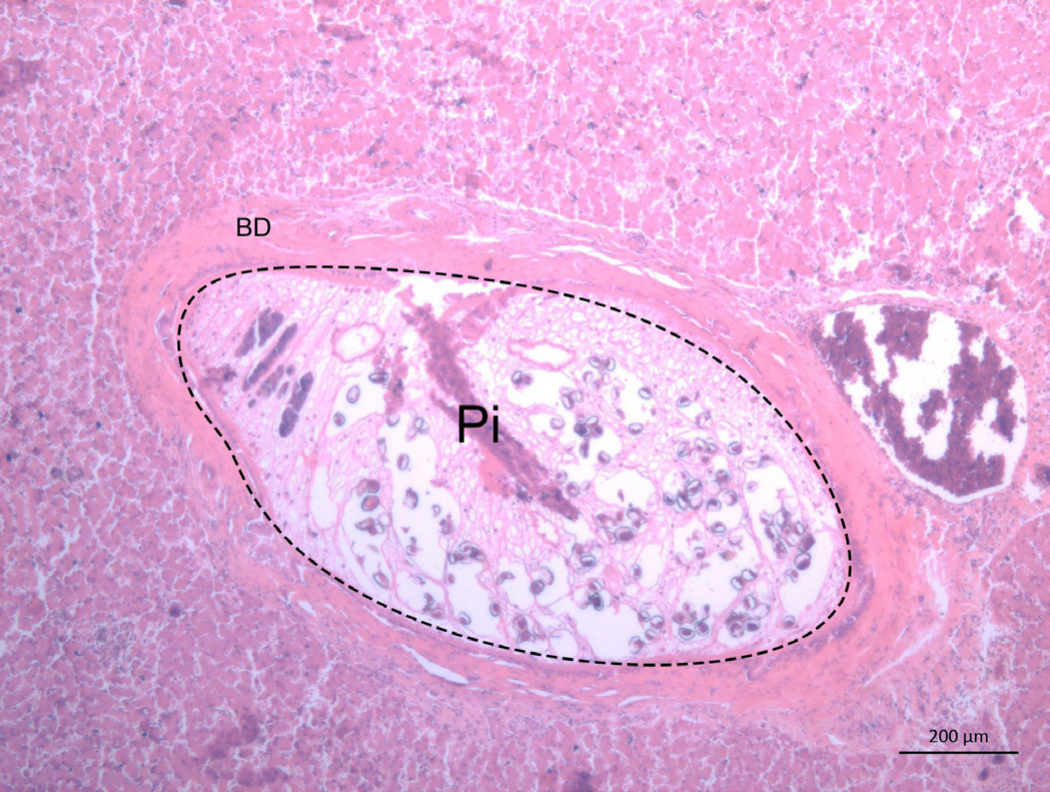
Photomicrograph of the liver of a cat naturally infected with *Platynosomum illiciens*. Cross sections of the parasite (Pi) inside a dilated and fibrous bile duct (BD). HE, 5X Objective.

A significant correlation between hepatic lesions and cachexia with *P. illiciens* was detected by GLM analysis (Table 2). Indeed, hepatic lesions were observed in 90.9% (10/11) of the necropsied cats parasitized with *P. illiciens*. These lesions consisted of hepatomegaly, whitish coloration, firm texture of the portal areas, thickness of bile ducts, and fibrosis. Microscopically, we observed cholangioectasis and *P. illiciens* inside the fibrotic and dilated bile ducts (Figure 1).

**Figure 2:**
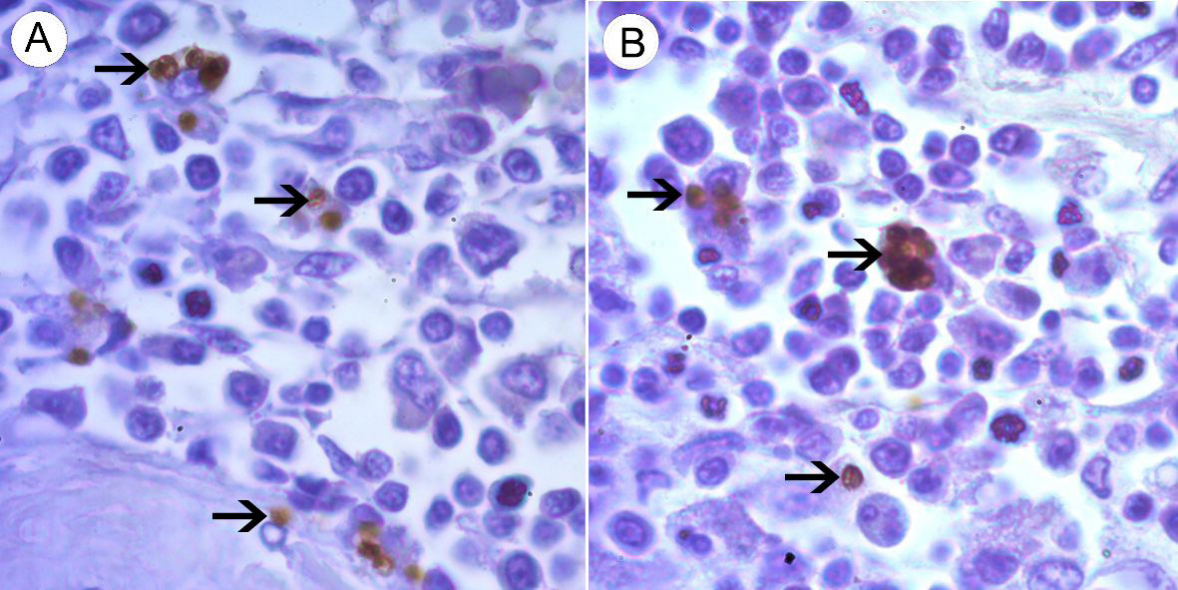
Photomicrograph of immunohistochemistry of spleen (A) and lymph node (B) of a cat infected with feline immunodeficiency virus, *Toxoplasma gondii*, *Leishmania* spp., and *Platynosomum illiciens*. Note the amastigote forms (arrows) in the cytoplasm of macrophages and in extracellular space. IHC, 100X Objective.

**Figure 3:**
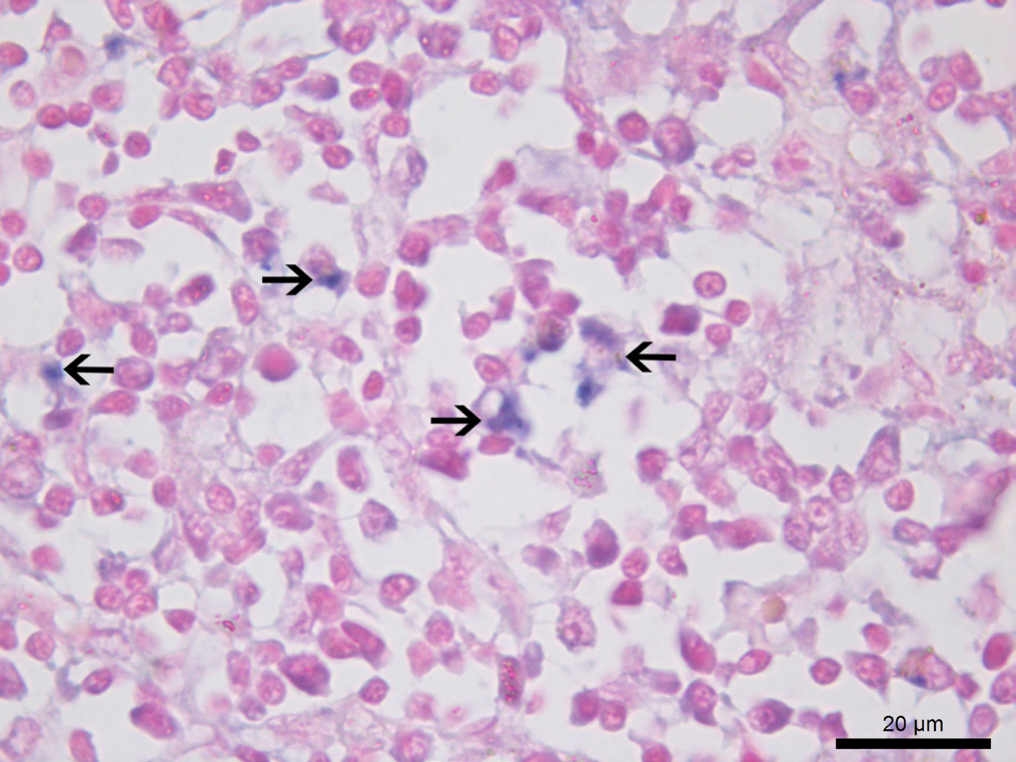
Photomicrograph of the lymph node of a cat coinfected with feline immunodeficiency virus, *Toxoplasma gondii*, *Leishmania infantum,* and *Platynosomum illiciens*. Chromogenic *in situ* hybridization showing blue-stained amastigote forms of *L. infantum* (arrows) inside the cytoplasm of macrophages. CISH, 100X Objective.

**Figure 4:**
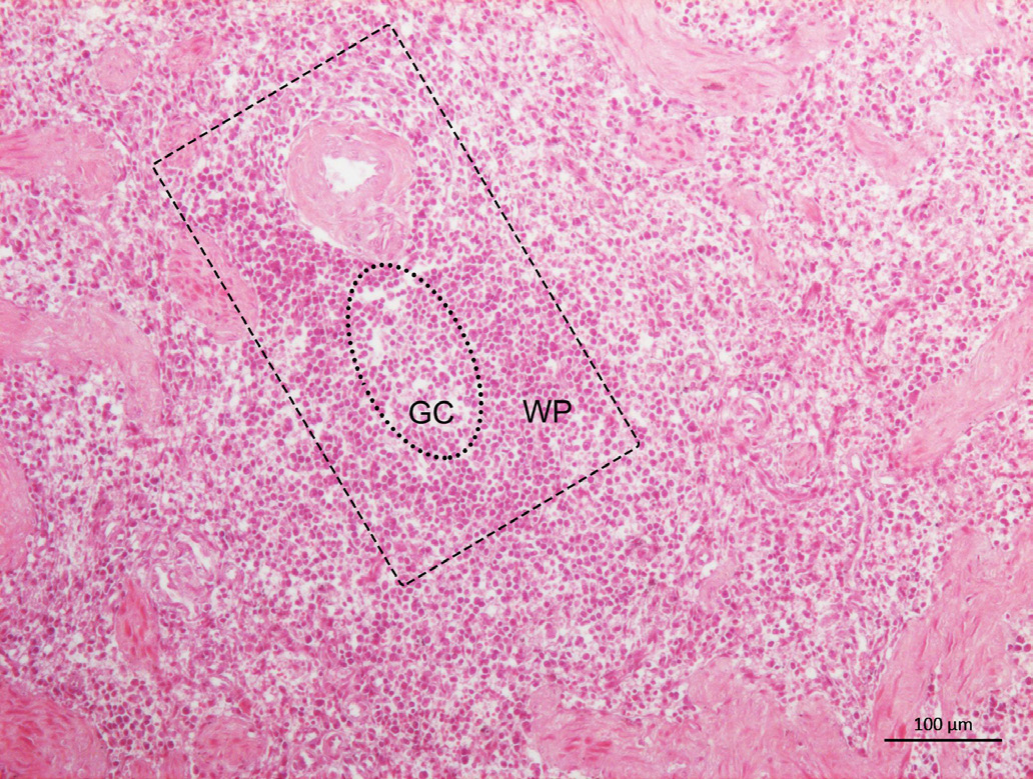
Photomicrograph of a spleen of a cat coinfected with feline immunodeficiency virus, *Toxoplasma gondii*, *Leishmania infantum,* and *Platynosomum illiciens*. Note the disorganized white pulp (WP, rectangle) with hypoplastic germinal center (GC, ellipse). HE, 10X Objective.

**Figure 5:**
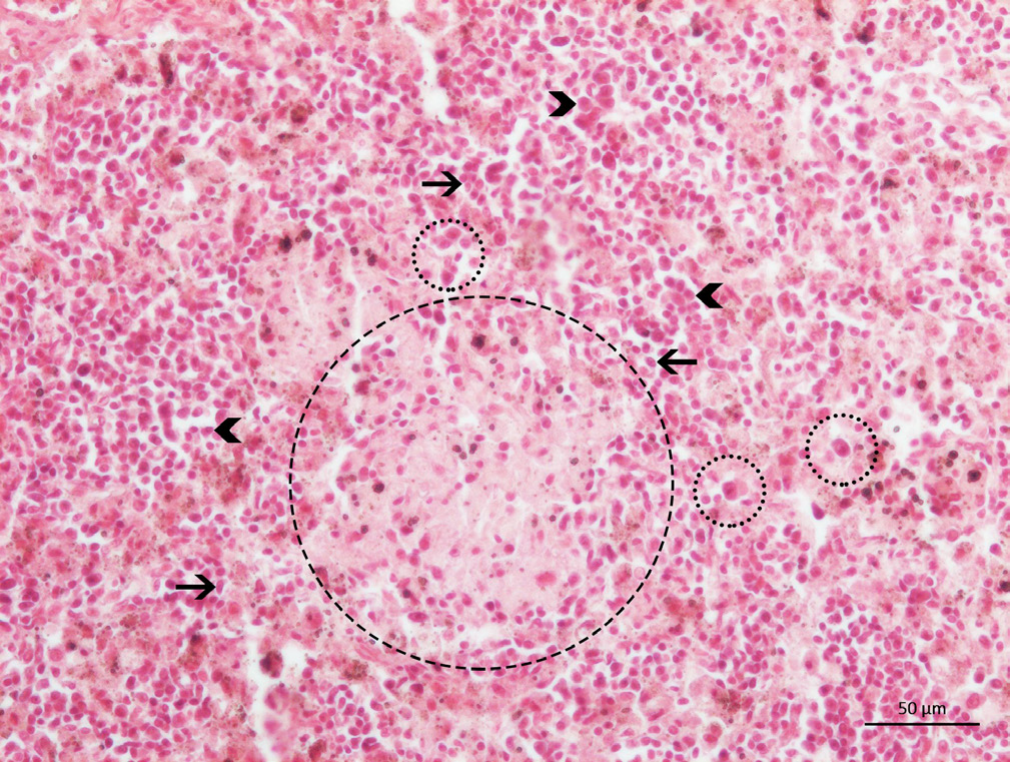
Photomicrograph of a lymph node of a cat coinfected with feline immunodeficiency virus, *Toxoplasma gondii*, *Leishmania infantum,* and *Platynosomum illiciens*. Note the formation of granuloma (large circle) with the central area of necrosis and the presence of plasma cells (head arrows), histiocytes (small circles) and epithelioid cells (arrows). HE, 20X Objective.

Among the *Leishmania* spp. seropositive cats (n=5), the IHC detected amastigote forms of *Leishmania* spp. inside the macrophages, as well as diffused in the interstitial space of the spleen and lymph nodes of a single animal coinfected with *P. illiciens*, *T. gondii*, and FIV (Figure 2A and 2B). In addition, using CISH, infection with *L. infantum* was confirmed in this animal (Figure 3). Furthermore, the histopathological analysis of this polyparasitized cat demonstrated great disorganization of the white pulp together with the atrophy of the germinal center (Figure 4), with marginal zone and lymphoid follicle atrophy. In the red pulp, histiocytosis, epithelioid cells and Mott cells were also noted. Also, we observed granulomas, plasmacytosis, histiocytosis, and epithelioid cells in the lymph node parenchyma (Figure 5).

## Discussion

Although the seroprevalence of 10% observed here was greater than that found by Braga et al. (2014a) and Metzdorf et al. (2017) in cats from Campo Grande, Mato Grosso do Sul state, the role of this domestic species in the transmission and maintenance of *L. infantum* in endemic areas of Brazil is not well established (Nascimento et al., 2022). Cats seem to have no epidemiological importance in endemic areas for VL since we found only one animal with *L. infantum*, in accordance with Berenguer et al. (2021) that detected amastigotes forms in a single cat (1/128) from Pernambuco state. Furthermore, Braga et al. (2014b) did not show amastigotes forms in seropositive cats (15/50) from Campo Grande. Moreover, the infection by *L. infantum* in cats has demonstrated minimal or limited pathological changes (Solano-Gallego et al., 2007; Pennisi et al., 2015; Soares et al., 2016), in agreement with our results which showed no correlation between *Leishmania* spp. seropositive cats and gross lesions.

The presence of amastigote forms of *L. infantum* in the single cat may be related to the coinfection by *T. gondii*, *P. illiciens*, and FIV. Indeed, the immunosuppression caused by retrovirus may facilitate parasite survival in macrophages by evading natural killer cells and cytotoxic lymphocyte activity (Dorny et al., 2002; Grevot et al., 2005; Sobrinho et al., 2012; Spada et al., 2012). In fact, the parasite persistence during coinfection by *L. infantum* and retroviruses was reported in humans as a consequence of the impairment of macrophage function (Rossi & Fasel, 2018) and probably contributed to the lymphoid tissue damage recorded in the cat with amastigotes forms of *L. infantum*, also observed in severe canine VL with high parasite load (Cavalcanti et al., 2015). Furthermore, histiocytosis and Mott cells observed in the spleen, as well as increase in plasma cells and granulomas in the lymph node of this animal, suggest a chronic infection (Moreira et al., 2010; Mossuto et al., 2015; Lucas, 2017). The plasmacytosis observed in the lymph node of the cat coinfected by *L. infantum, T. gondii*, *P. illiciens*, and FIV is a common finding related to VL (Veress et al., 1977; Campos-Neto & Bunn-Moreno, 1982; Galvão-Castro et al., 1984; Bagues et al., 2018; Silva-Barrios et al., 2018), and in lentiviral infections (Takano et al., 2012; Magden et al., 2013).

The polyparasitism was demonstrated in 51.8% (14/27) of the infected cats, in accordance with several reports in the studied area (Braga et al., 2014a; Sousa et al., 2014; André et al., 2015), in Brazilian semiarid region (Miró et al., 2014; Feitosa et al., 2021), and worldwide (Poli et al., 2002; Ayllón et al., 2012; Sobrinho et al., 2012; Akhtardanesh et al. 2020). Sobrinho et al. (2012) showed a strong association between *Leishmania* sp., FIV, and *T. gondii* in naturally infected cats in an endemic area for VL in southwest Brazil. Additionally, Feitosa et al. (2021) reported the correlation between FIV and *T. gondii* in owned cats of Brazilian semiarid region. However, the outcomes of coinfections on the host environment are still largely neglected.

Our analysis demonstrated that cachexia and hepatopathy were significantly related to parasitism by *P. illiciens¸* helminth parasite found in all coinfected cats. Liver impairment as a consequence of chronic cholangiohepatitis observed here has been reported in platynosomiasis (Headley et al., 2012; Jesus et al., 2015; Ramos et al., 2017) and certainly compromises the health of their hosts favoring coinfections. Similarly, *T. gondii* was found in 57.1% of coinfected cats, and when associated to *L. infantum*, FIV and/or FeLV may constitute a risk for the health of coinfected animals (Sobrinho et al., 2012; Feitosa et al., 2021). In fact, although protozoan infection primarily induces a strong immune response, it subsequently fails to clear the infection, leading to a chronic phase (Gigley et al., 2012). In the case of coinfection with FIV and *T. gondii*, clinical and fatal systemic toxoplasmosis have been recorded (Davidson et al., 1993), analogous to the reports in people with toxoplasmosis and human immunodeficiency virus (Xavier et al., 2013).

Although anemia has long been related to FeLV (Hartmann, 2011; Cavalcante et al., 2018; Abdollahi-Pirbazari et al., 2019; Akhtardanesh et al., 2020; Gomez-Lucia et al., 2020), we found a strong correlation between FIV and low RBC, as reported by Spada et al. (2012), suggesting that FIV may compromise the hematopoietic tissues. The association between splenomegaly and FeLV observed in the sampled cats may be linked to extramedullary hematopoiesis response to anemia, described in the late phases of FeLV infection (Shirani et al., 2011; Hartmann, 2012). The immunosuppression frequently detected in FeLV predisposes to secondary infections resulting in lymphadenomegaly (Quackenbush et al., 1996; Hartmann, 2012; Hartmann & Hofmann-Lehmann, 2020), a condition significatively correlated to FeLV seropositive cats in the present study. Furthermore, monocytosis strongly associated with FeLV were also reported in chronic myelomonocytic leukemia with FeLV infection by Shimoda et al. (2000). We highlight that splenomegaly and lymphadenomegaly in 88.9% of cats seropositive for FeLV should be considered an important indicator of this infection.

## Conclusions

This study reported that coinfections by *L. infantum*, *T. gondii*, and *P. illiciens* result in damage to lymphoid tissues and liver, threatening the health of infected animals. Polyparasitism in stray cats of the Brazilian Midwest should be considered in order to assure effective control strategies for public health diseases. Moreover, stray cats of Campo Grande may be a source of infection of FIV, FeLV and *P. illiciens* for populations of domiciled cats.
